# Dysbiosis of oral microbiome persists after dental treatment-induced remission of periodontal disease and dental caries

**DOI:** 10.1128/msystems.00683-23

**Published:** 2023-09-12

**Authors:** Kazuma Yama, Yuichiro Nishimoto, Kota Kumagai, Ryutaro Jo, Minori Harada, Yuki Maruyama, Yuto Aita, Narumi Fujii, Takuya Inokuchi, Ryosuke Kawamata, Misato Sako, Yuko Ichiba, Kota Tsutsumi, Mitsuo Kimura, Shinnosuke Murakami, Yasushi Kakizawa, Takashi Kumagai, Takuji Yamada, Shinji Fukuda

**Affiliations:** 1 Research and Development Headquarters, Lion Corporation, Tokyo, Japan; 2 Metagen Inc., Kakuganji, Tsuruoka, Yamagata, Japan; 3 Hiyoshi Oral Health Clinics, Sakata, Yamagata, Japan; 4 Institute for Advanced Biosciences, Keio University, Kakuganji, Tsuruoka, Yamagata, Japan; 5 Department of Life Science and Technology, Tokyo Institute of Technology, Meguro-ku, Tokyo, Japan; 6 Gut Environmental Design Group, Kanagawa Institute of Industrial Science and Technology, Kawasaki-ku, Kawasaki, Kanagawa, Japan; 7 Transborder Medical Research Center, University of Tsukuba, Tsukuba, Ibaraki, Japan; 8 Laboratory for Regenerative Microbiology, Juntendo University Graduate School of Medicine, Bunkyo-ku, Tokyo, Japan; Oregon State University, Corvallis, Oregon, USA

**Keywords:** oral microbiome, periodontal disease, dental caries, metabolome, dysbiosis

## Abstract

Oral diseases, such as periodontal disease and dental caries, have a high risk of recurrence and are considered to be related to dysbiosis of the oral microbiome and metabolome. It is, therefore, important to understand the state of the oral microbiome and metabolome after disease treatment to prevent recurrence. The current study sought to clarify whether oral dysbiosis improves the following remission of symptoms by dental treatment. To this end, the salivary microbiome and metabolome of healthy participants were compared to those with periodontal disease, dental caries, or both (concurrent). Saliva was collected as mouth-rinsed water and the microbiome was measured using 16S rRNA gene targeted sequencing and metabolome using capillary electrophoresis–time-of-flight mass spectrometry. Comparisons with healthy participants were performed before and after treatment, and several months after transition to self-care. Dental treatment significantly improved the oral health condition of each group; several months after treatment, oral health did not deteriorate. However, even after remission, the salivary microbiome of the two groups (oral disease and healthy) differed significantly. Additionally, after remission, significant differences, compared with healthy participants, remained in the relative abundances of disease-related bacteria and nitrate-reducing bacteria. After remission, significant differences were observed in the salivary metabolome in the healthy group in terms of threonate and pyrimidine metabolism-related component concentrations, which are assumed to reflect the high relative abundance of periodontal disease-related bacteria in the microbiome. Oral microbiome dysbiosis persisted even after dental treatment-induced disease remission with a sustained increased risk of disease when compared with healthy participants.

**IMPORTANCE:**

We characterized the oral conditions, salivary microbiome, and metabolome after dental treatment by investigating the state after treatment completion and transition to self-care. Dental treatment improved oral health conditions, resulting in oral disease remission; however, the imbalanced state of the salivary microbiome continued even after remission. Although the results of this study are preliminary, owing to the small number of participants in each group when compared to larger cohort studies, they indicate that the risk of disease may remain higher than that of healthy participants, thereby demonstrating the importance of removing dental plaque containing disease-related bacteria using appropriate care even after treatment completion. We also identified bacterial species with relative abundances that differed from those of healthy participants even after remission of symptoms, which may indicate that the maturation of certain bacterial species must be controlled to improve the oral microbiome and reduce the risk of disease recurrence.

## INTRODUCTION

Periodontal disease and dental caries are highly prevalent worldwide and constitute a major health issue (1, 2). Moreover, these diseases are likely to recur following initial onset and remission (3, 4) and are caused by caries-related bacteria (e.g., Mutans streptococci*, Bifidobacterium*), which have acid-producing and -resistant properties, and periodontal disease-related bacteria (e.g., red complex bacteria including *Porphyromonas gingivalis,* orange complex including *Prevotella intermedia* and *Filifactor alocis*) that proliferate in an anaerobic environment by obtaining iron from gingival bleeding (5 – 8). The onset of oral diseases is related to dysbiosis of the oral microbiome wherein the relative abundances of these disease-related bacteria are high (5, 6), and to changes in metabolites, such as lactate production during dental caries and pyrimidine metabolism-related components during periodontal disease (9 – 11). Hence, understanding the oral microbiome and metabolome status after treatment (AT) is necessary to prevent disease recurrence.

Treatment of periodontal disease-related gingival inflammation primarily involves removal of dental plaque, which may cause inflammation. Although basic periodontal treatment reduces the abundance of periodontal disease-related bacteria within the subgingival margin (12, 13), the salivary microbiome of patients after one basic periodontal treatment differs from that of healthy participants (14). Hence, periodontal disease-related bacteria may remain in the saliva even after the periodontal area is cleaned and subgingival dental plaque is removed. However, whether the salivary microbiome and metabolites differ from healthy participants after completion of treatment (CT) and remission of symptoms remains unclear.

Dental caries, the etiology of which differs from that of periodontal disease (6, 15), are removed by extracting or scraping. However, as with periodontal disease, the state of the salivary microbiome and metabolites AT is unknown. Therefore, the primary aim of this study was to clarify whether salivary microbiome/metabolome profiles approach a healthy state or remain different after dental treatment-associated disease remission. To this end, saliva samples were collected as mouth-rinsed water, a method with low subject burden (16, 17), and the salivary microbiome and metabolome profiles of patients with periodontal disease, dental caries, or both were compared to those of healthy participants before treatment (BT), at CT, and several months after CT following transition to self-care (AT).

## RESULTS

### Oral health conditions of the healthy and disease groups

**Fig 1:**
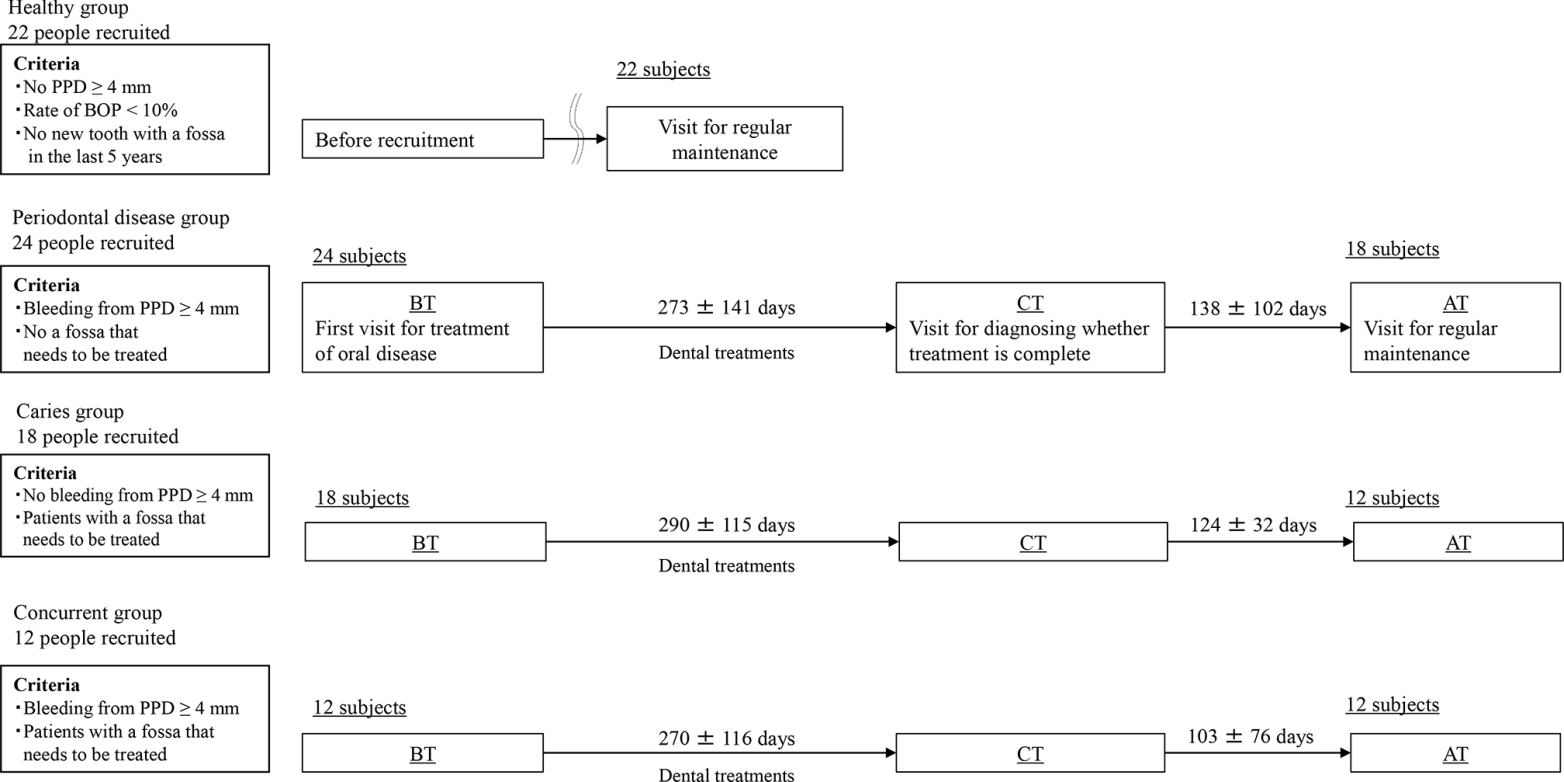
Study flowchart. Criteria and sampling periods are presented. Abbreviations: BT, before treatment; CT, completed treatment. AT, after treatment; PPD, probing pocket depth; BOP, bleeding on probing.

We recruited 22 participants who had no newly diagnosed dental caries over the past 5 years and no probing pocket depth (PPD) ≥4 mm, as the healthy group and 54 patients who had visited the dental clinic for treatment, as the disease group. The disease group was further categorized based on oral examination results into the periodontal disease, caries, and concurrent (caries and periodontal disease) groups. Saliva samples from the healthy group were collected when the participants visited the clinic for regular maintenance, and saliva samples from the disease group were collected when the participants visited the clinic BT, at CT, and AT (123 ± 81 d after remission; mean ± standard deviation [SD]); three samples per participant (Fig. 1). The microbiome and metabolite profiles of patients who had completed sample collection until AT were included in the analysis (periodontal disease: *n =* 16; caries: *n =* 12; concurrent: *n* = 12), except for two samples, which had contamination and dilution concerns. There were no significant differences in the age or number of remaining teeth between the healthy and disease groups BT (Table 1). The caries and concurrent groups had decayed teeth BT. Additionally, the number of decayed and filled teeth were higher in all disease groups than in the healthy group. The average PPD did not differ significantly between the healthy group and each disease group. However, sites wherein the PPD was ≥4 mm—considered a clinical indicator of periodontal disease (18)—were not present in the healthy group, and were present in approximately 10% of each disease group. The proportion of bleeding on probing (BOP), when compared to that in the healthy group, was significantly higher in the periodontal disease and concurrent groups, and significantly lower in the caries group. There were no decayed teeth that required treatment in the caries and concurrent groups at CT. Periodontal treatment improved the periodontal condition, and the average PPD and proportion of PPD ≥ 4 mm were significantly lower at CT and AT than BT in all disease groups. The proportion of BOP was significantly improved in the periodontal disease and concurrent groups at CT when compared to that at BT, and no significant differences were observed between the concurrent and healthy groups at AT. No significant differences were found in the total bacterial concentration in mouth-rinsed water samples between the healthy and each disease group BT at CT, and AT.

**TABLE 1:** Age, oral examination results, and total bacterial concentration of participants in the healthy and disease groups

	Group									
	Healthy	Periodontal disease			Caries			Concurrent		
		BT^*e*^	CT^*f*^	AT^*g*^	BT^*e*^	CT^*f*^	AT^*g*^	BT^*e*^	CT^*f*^	AT^*g*^
Number of patients (F/M)*^h^* ^,^ *^i^*	22 (12/10)	16 (11/5)	−	−	12 (7/5)	−	−	12 (6/6)	−	−
Age (mean ± SD)	43.7 ± 11.8	49.0 ± 13.4	−	−	42.4 ± 11.4	−	−	48.9 ± 10.7	−	−
Number of teeth	28.0 ± 1.1	27.8 ± 2.7	27.2 ± 2.8	26.9 ± 3.0^*c*^	28.8 ± 1.7	27.7 ± 1.3^*a*^ ^,^ ^*c*^	27.7 ± 1.3*^a^* ^,*c*^	27.3 ± 2.7	27.1 ± 2.6	26.8 ± 2.4^*b*^
Number of DT **^*j*^**	0 ± 0	0 ± 0	0 ± 0	0 ± 0	2.5 ± 2.7^*b*^	0 ± 0^*d*^	0 ± 0^*d*^	2.0 ± 2.1^*b*^	0.2 ± 0.4^*d*^	0.2 ± 0.4^*d*^
Number of DFT **^*k*^**	6.5 ± 6.2	12.2 ± 6.8^*b*^	12.1 ± 6.7^*b*^	11.7 ± 6.2^*a*^	11.8 ± 6.6^*a*^	11.8 ± 6.6^*a*^	11.8 ±6.6^*a*^	12.8 ± 4.8^*b*^	13.0 ± 4.1^*b*^	13.1 ± 3.9^*b*^
Average PPD **^*l*^**	2.6 ± 0.3	3.0 ± 0.8	2.2 ± 0.6*^b^* ^,^ *^d^*	1.9 ± 0.5*^b^* ^,^ *^d^*	2.8 ± 0.6	2.3 ± 0.4*^a^* ^,^ *^d^*	2.3 ± 0.3*^b^* ^,^ *^c^*	2.9 ± 0.4^*b*^	2.3 ± 0.4^*d*^	2.2 ± 0.3*^b^* ^,^ *^d^*
PPD ≥4 mm (%)	0 ± 0	17.7 ± 13.4^*b*^	5.3 ± 7.3^*b*^ ^,^ *^d^*	3.0 ± 4.0*^b^* ^,^ *^d^*	9.8 ± 11.7^*b*^	2.7 ± 4.0*^b^* ^,*d*^	2.2 ± 3.2^*b*^ ^,^ ^*d*^	13.3 ± 12.6^*b*^	5.0 ± 7.8^*b*^ ^,^ ^*c*^	4.5 ± 6.4^*b*^ ^,^ ^*d*^
BOP (%)^*m*^	2.2 ± 2.1	40.2 ± 23.8^*b*^	11.4 ± 11.2^*b*^ ^,^ ^*d*^	11.2 ± 6.9*^b^* ^,^ *^d^*	1.4 ± 4.2^*a*^	0.5 ± 1.3^*a*^	1.0 ± 2.6^*a*^	39.0 ± 21.5^*b*^	9.8 ± 11.1^*d*^	5.8 ± 9.5^*d*^
BOP and PPD ≥4 mm (%)	0 ± 0	15.6 ± 12.4^*b*^	3.6 ± 6.8*^b^* ^,*d*^	1.9 ± 3.1*^b^* ^,^ *^d^*	0 ± 0	0 ± 0	0 ± 0	12.6 ± 12.2^*b*^	2.0 ± 3.1*^b^* ^,^ *^d^*	1.0 ± 2.0*^a^* ^,*d*^
Log_10_ total bacterial concentration (estimated CFU/mL)	8.64 ± 0.65	8.59 ± 0.81	8.73 ± 0.62	8.78 ± 0.53	8.54 ± 0.79	8.54 ± 0.36	8.75 ± 0.49	8.70 ± 0.72	9.12 ± 0.54	9.10 ± 0.69

^*a*^ Significantly different from the healthy group by Wilcoxon rank-sum test: *P* < 0.05.

^*b*^ Significantly different from the healthy group by Wilcoxon rank-sum test: *P* < 0.01.

^*c*^ Significantly different from BT by Wilcoxon signed-rank test: *P* < 0.05.

^*d*^ Significantly different from BT by Wilcoxon signed-rank test: *P* < 0.01.

^*e*^ BT, before treatment.

^*f*^ CT, completed treatment.

^*g*^ AT, after treatment.

^*h*^ F, female.

^*i*^ M, male.

^*j*^ DT, decayed tooth.

^*k*^ DFT, decayed and filled tooth.

^*l*^ PPD, probing pocket depth.

^*m*^ BOP, bleeding on probing.

### Treatment-induced changes in the oral microbiome

**Fig 2:**
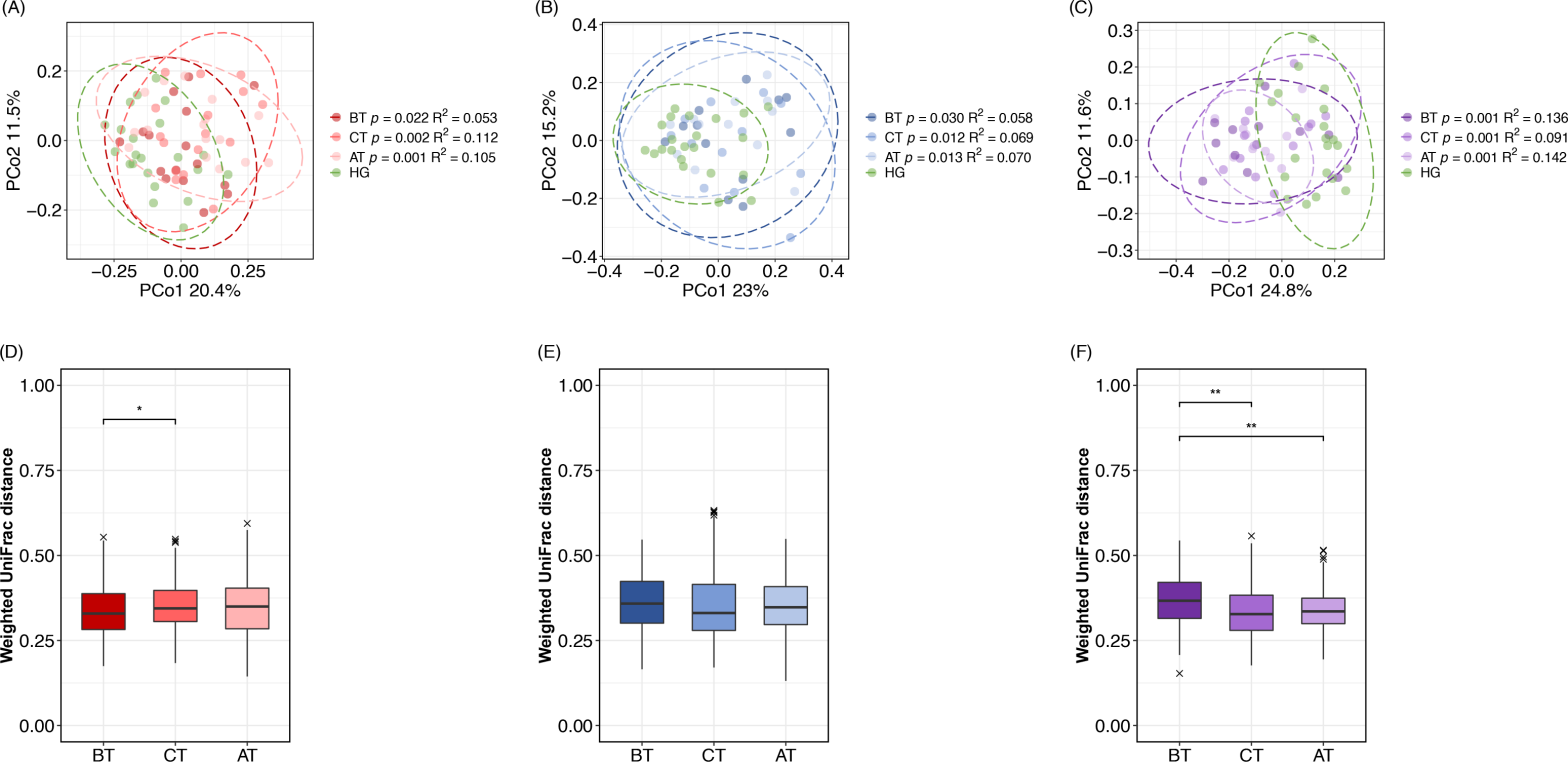
Principal coordinate analysis (PCoA) plot of the oral microbiome in the healthy group (HG) and each disease group based on weighted UniFrac distance. The ellipses indicate multivariate *t*-distribution with a confidence interval of 0.95. (**A**) PCoA plot of the healthy and periodontal disease groups. (**B**) PCoA plot of the healthy and caries groups. (**C**) PCoA plot of the healthy and concurrent groups. The *P-*values and *R* ^2^ values of the PERMANOVA test results for each disease group when compared to the healthy group at each sampling time point are listed in the figure keys. The ANOSIM test results are also listed in Table S4. (D–F) Changes in the weighted UniFrac distance from the healthy group to disease group at each sampling point. Steel–Dwass test *P*-value was used to determine statistical significance. **P < 0.01, *P < 0.05. (**D**) Distance transition between the healthy and periodontal disease groups from before treatment (BT) to after treatment (AT). (**E**) Distance transition between the healthy and caries groups from BT to AT. (**F**) Distance transition between the healthy and concurrent groups from BT to AT.

We investigated whether the oral microbiome of each disease group BT and AT differed from that of healthy participants by comparing the salivary microbiome of each disease group BT, at CT, and AT with that of the healthy group using alpha-diversity and the weighted UniFrac distance. The number of operational taxonomic units (OTUs) was significantly higher in all disease groups when compared with the healthy group BT and it remained significantly higher in the periodontal disease and concurrent groups when compared with the healthy group AT. The Shannon diversity index was significantly higher in the periodontal and concurrent groups than in the healthy group BT, and significantly higher in the concurrent group AT (Table 2). The oral microbiome profiles of the healthy group and all disease groups BT, at CT, and AT differed significantly as shown in Fig. 2A, B, and C using principal coordinate analysis (PCoA) with weighted UniFrac distance. Furthermore, the *R* ^2^ values of the PERMANOVA test showed that the highest values were obtained when comparing the healthy and concurrent groups (vs periodontal disease group: *R* ^2^ = 0.053; vs caries group: *R* ^2^ = 0.058; vs concurrent group: *R* ^2^ = 0.136). Next, we investigated whether the treatment caused the microbiome to approach a healthier state by comparing the weighted UniFrac distances of each disease group with that of the healthy group BT, at CT, and AT (Fig. 2D, E and F). No significant differences were observed between the periodontal disease/caries group and healthy groups BT, at CT, and AT. Meanwhile, the distance from the concurrent group to the healthy group significantly decreased at CT and AT when compared to that of BT, thereby becoming more similar to the healthy group.

**TABLE 2:** Alpha diversity of the healthy and disease groups

	Group									
	Healthy	Periodontal disease			Caries			Concurrent		
		BT^*e*^	CT^*f*^	AT^*g*^	BT^*e*^	CT^*f*^	AT^*g*^	BT^*e*^	CT^*f*^	AT^*g*^
Observed OTUs	173 ± 38	227 ± 57^*b*^	211 ± 43^*b*^	206 ± 40*^a^* ^,*c*^	223 ± 46^*b*^	196 ± 31	202 ± 42	214 ± 55^*a*^	207 ± 74	207 ± 42^*a*^
Shannon diversity	3.31 ± 0.29	3.60 ± 0.34^*a*^	3.54 ± 0.28^*a*^	3.48 ± 0.27	3.53 ± 0.39	3.34 ± 0.45	3.46 ± 0.37	3.55 ± 0.37^*a*^	3.58 ± 0.43	3.61 ± 0.22^*b*^

^*a*^ Significantly different from the healthy group by Wilcoxon rank-sum test: *P* < 0.05.

^*b*^ Significantly different from the healthy group by Wilcoxon rank-sum test: *P* < 0.01.

^*c*^ Significantly different from BT by Wilcoxon signed-rank test: *P* < 0.05.

^*d*^ Significantly different from BT by Wilcoxon signed-rank test: *P* < 0.01.

^*e*^ BT, before treatment.

^*f*^ CT, completed treatment.

^*g*^ AT, after treatment.

### Treatment-induced changes in bacteria relative abundances

**Fig 3:**
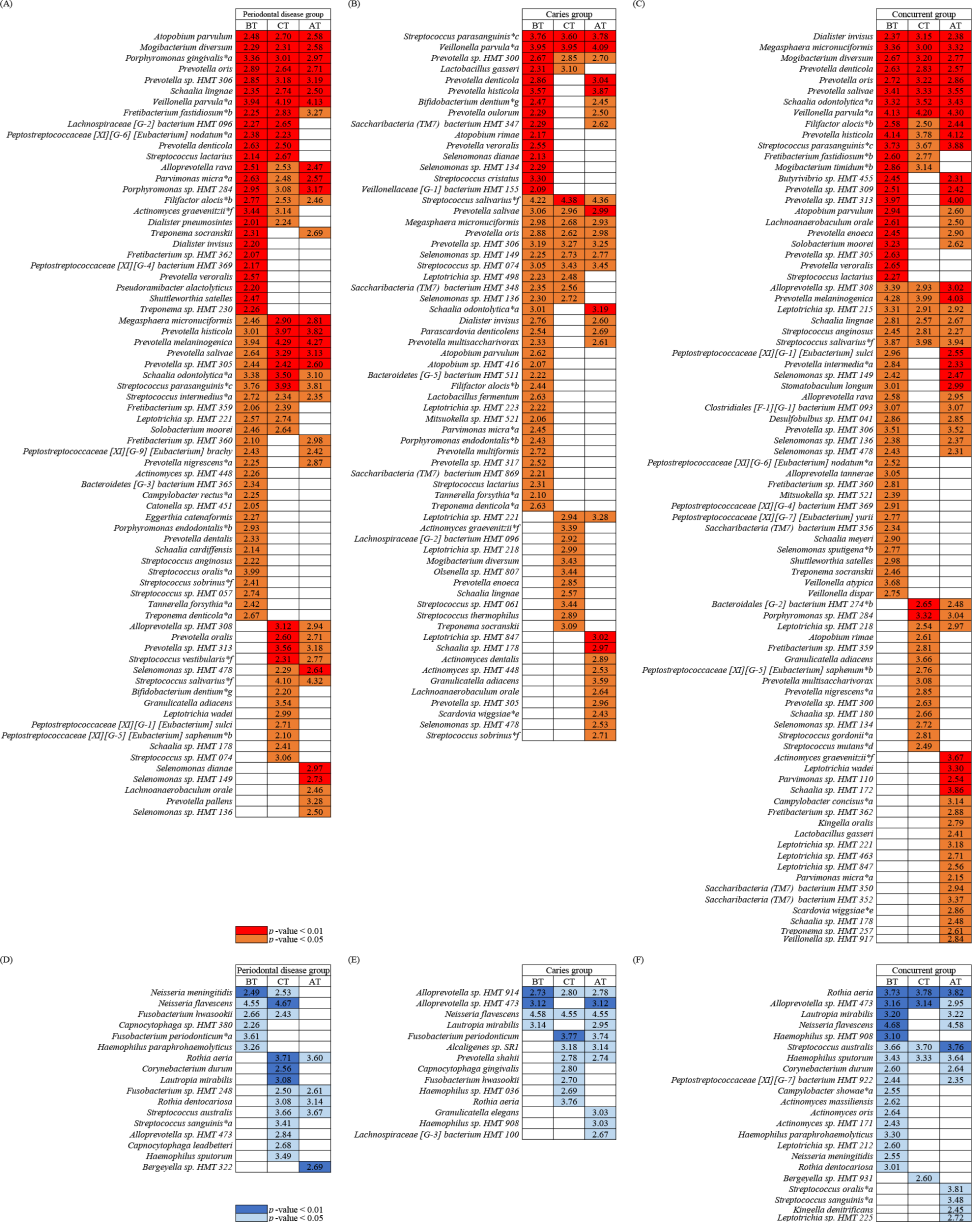
(**A, B and C**) Heat map of bacteria with a significantly higher relative abundance in the disease group than in the healthy group. Bacterial species with a significantly different abundance from those in the healthy group were selected based on the *P*-values (<0.05) of the Wilcoxon rank-sum test and LDA score (>2) and *P*-values (<0.05) of the linear discriminant analysis effect size (LEfSe). Wilcoxon rank-sum test *P*-values < 0.05 are shown in orange, and those <0.01 are shown in red. The value in the square indicates the Log_10_ value of the LDA score. The results of the two tests and the mean relative abundance and prevalence of each species are shown in Table S6. (**A**) Comparison between the periodontal disease and healthy groups; (**B**) comparison between the caries and healthy groups; (**C**) comparison between the concurrent and healthy groups. (**D, E and F**) Heat map of bacterial species with significantly higher relative abundances in the healthy group than in the disease groups. Wilcoxon rank-sum test *P*-values < 0.05 are shown in light blue, and those <0.01 are shown in blue. (**D**) Comparison between the periodontal disease and healthy groups; (**E**) comparison between the caries and healthy groups; (**F**) comparison between the concurrent and healthy groups. Bacterial species found to be related to each disease in previous reports are indicated by *a–g. Bacterial species a–c have been suggested to be related to periodontal disease (6 – 8, 20). Bacterial species d–g have been suggested to be related to dental caries (6, 21 – 24).

We focused on bacterial species with significant differences in relative abundances between the healthy and disease groups BT, at CT, and AT to investigate bacterial species with different relative abundances in the healthy state. Bacterial species with significantly different abundance from that in the healthy group were selected based on the *P*-values (<0.05) of the Wilcoxon rank-sum test and LDA score (>2) and *P*-values (<0.05) of the linear discriminant analysis effect size (LEfSe) (19). Fig. 3A, B and C show bacterial species with significantly higher abundances in each disease group than in the healthy group. To evaluate whether dysbiosis (a state in which the abundances of disease-related bacteria in the salivary microbiome are high) continues after remission of symptoms, we labeled (with *a–g) species that were previously described as being related to periodontal disease and dental caries in reviews (6) and in their references (7, 8, 20 – 24). In the periodontal group (Fig. 3A), 54 species including periodontal disease-related bacteria were present in higher abundances than those in the healthy group BT, of which 20 species including *Porphyromonas gingivalis*, as red complex bacterium were significantly different from the healthy group, both at CT and AT. In the caries group (Fig. 3B), 44 species, including periodontal disease-related bacteria, were present in higher abundances than those in the healthy group BT, of which 10 species were significantly different from the healthy group both at CT and AT. In addition, 9 species including *Bifidobacterium dentium* were significantly different from the healthy group BT and AT. And *Scardovia wiggsiae* and *Streptococcus sobrinus* were significantly different from the healthy group AT. In the concurrent group (Fig. 3C), 52 species including periodontal disease-related bacteria were more abundant than those in the healthy group, of which 17 species including *Filifactor alocis* were significantly different from the healthy group at CT and AT. In addition, 17 species including *Prevotella intermedia* as orange complex bacterium were significantly different from the healthy group BT and AT. And *S. wiggsiae* was significantly different from the healthy group AT. In contrast, Fig. 3D, E and F shows that bacteria were less common in the disease group than in the healthy group, that is, more common in the healthy group. In all disease groups, species with significantly higher relative abundance in the healthy group were identified, including *Neisseria flavescens*. Furthermore, the genera *Haemophilus* and *Rothia*, which are similar to *Neisseria* and involved in nitrate reduction reactions in the oral cavity, were extracted as species with significantly higher abundance in the healthy group when compared with each disease group.

### Treatment-induced changes in the metabolome profile

**Fig 4:**
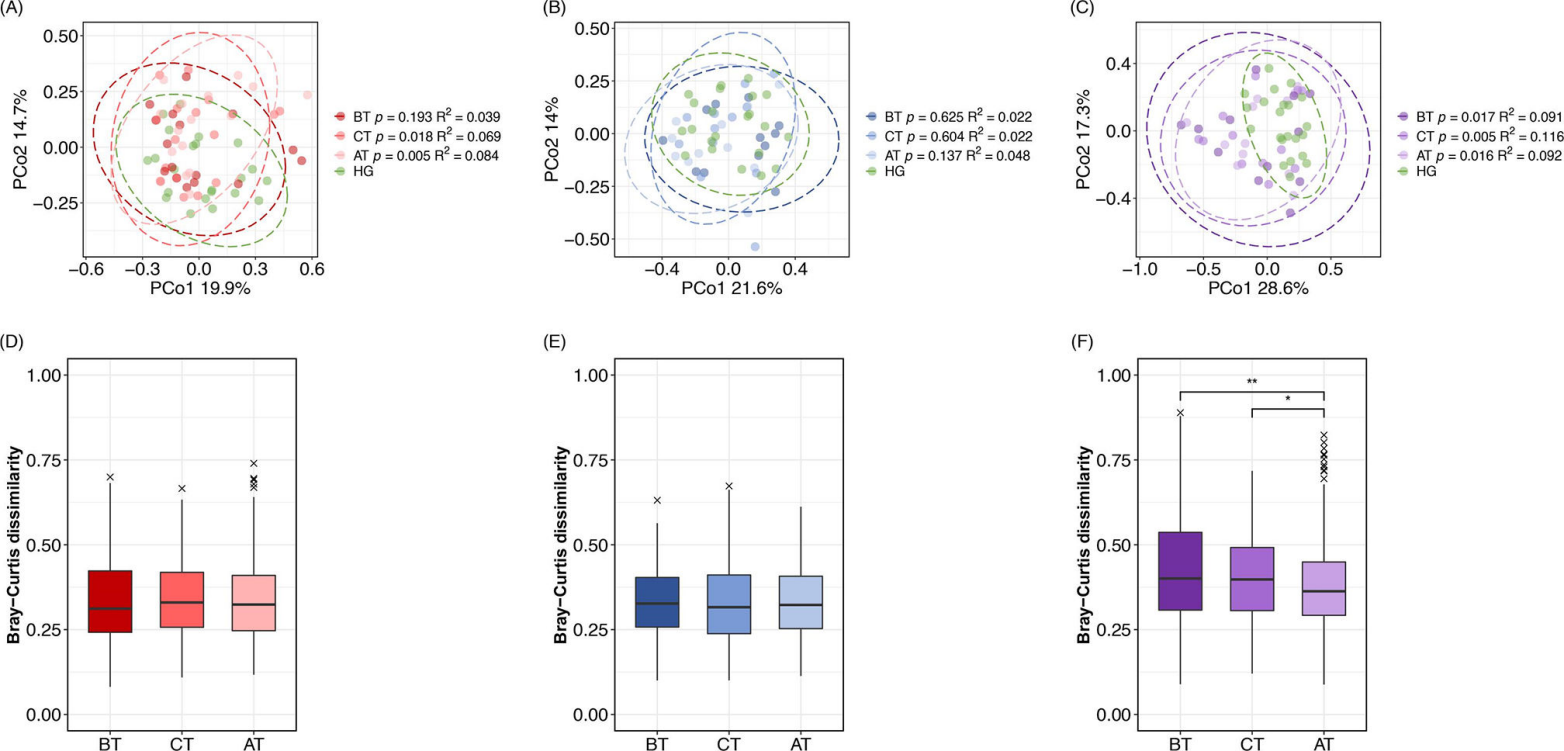
Principal coordinate analysis (PCoA) plot of the oral metabolome in the healthy (HG) and each disease group based on Bray–Curtis dissimilarity. The healthy group and each disease group are shown using different colors. The color lightens as the treatment progresses in the disease groups. The ellipses indicate multivariate *t*-distribution with a confidence interval of 0.95. (**A**) PCoA plot of the healthy and periodontal disease groups. (**B**) PCoA plot of the healthy and caries groups. (**C**) PCoA plot of the healthy and concurrent groups. The *P*-values and *R^2^* values of the PERMANOVA test for each disease group when compared to the healthy group at each sampling time point are listed in the keys. The ANOSIM test results are also listed in Table S8. (**D, E, F**) Changes in the Bray–Curtis dissimilarity with the healthy group during and after treatment in each disease group. The Steel–Dwass test *P*-value was used to determine statistical significance. **P < 0.01, *P < 0.05. (**D**) Dissimilarity transition between the healthy and periodontal disease groups from before treatment (BT) to after treatment (AT). (**E**) Dissimilarity transition between the healthy and caries groups from BT to AT. (**F**) Dissimilarity transition between the healthy and concurrent groups from BT to AT.

We investigated whether the oral metabolome profile in each disease group differed from that in the healthy group by comparing the salivary metabolome profiles of each group with those of the healthy group BT, at CT, and AT using PCoA with Bray–Curtis dissimilarity. No significant differences were observed between the healthy and periodontal disease groups BT. However, the metabolome profiles differed significantly at CT and AT (Fig. 4A). No significant differences in metabolome profiles were observed between the healthy and caries groups BT, at CT, or AT (Fig. 4B). Significant differences in metabolome profiles were observed between the healthy and concurrent groups BT, at CT, and AT (Fig. 4C). Next, we investigated whether the metabolome profiles approached that of a healthy state following treatment by comparing the Bray–Curtis dissimilarity of each disease group with that of the healthy group BT, at CT, and AT (Fig. 4D through F). No significant differences were observed in similarity between the healthy and periodontal disease or caries groups from BT to AT. Meanwhile, the similarity of the concurrent group with the healthy group was significantly higher AT than that of BT.

### Treatment-induced changes in the concentrations of individual metabolites

**Fig 5:**
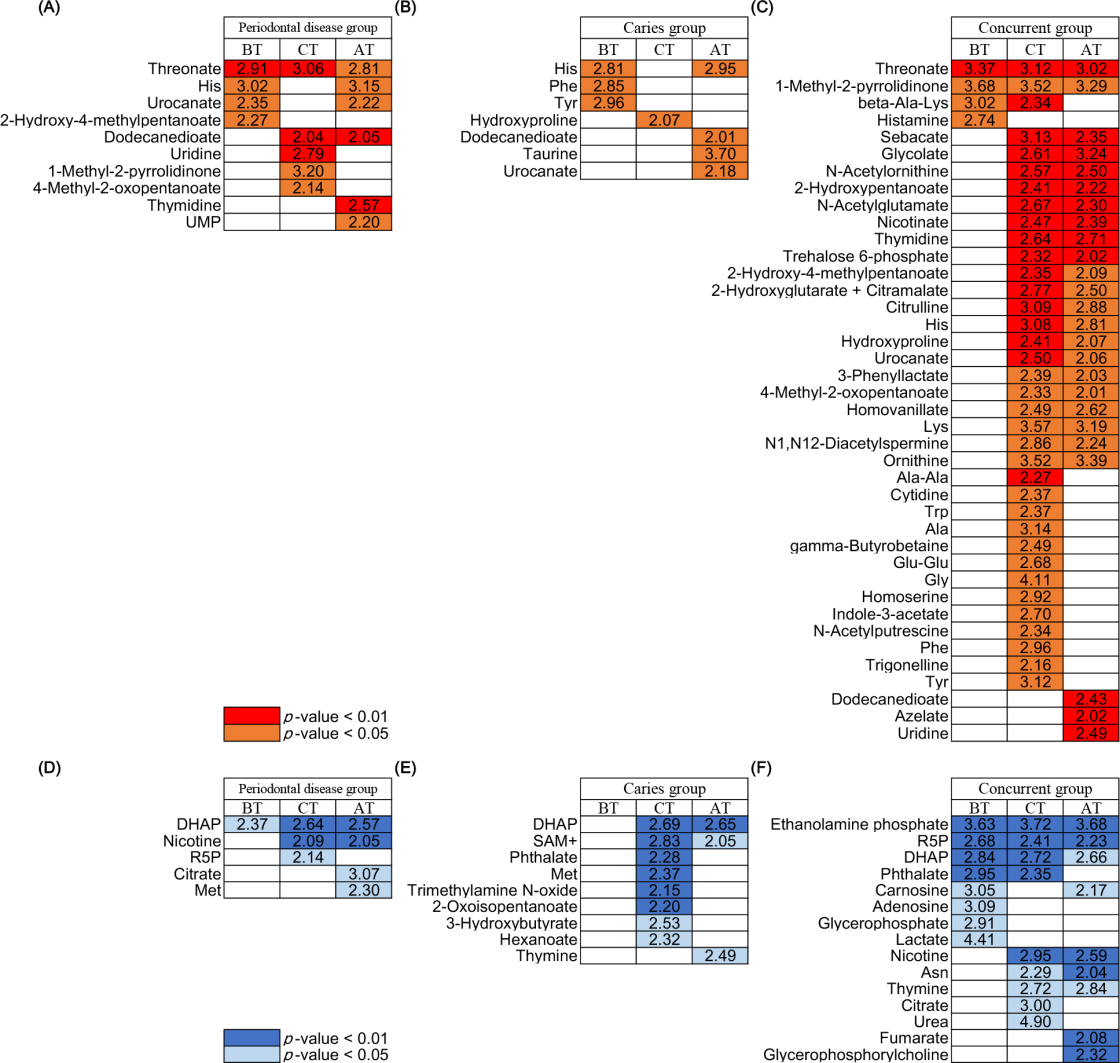
(**A, B and C**) Heat map of oral metabolites with significantly higher concentrations in each disease group when compared with the healthy group. Metabolites with significantly different concentrations from those in the healthy group were selected based on the *P*-values (<0.05) of the Wilcoxon rank-sum test and the LDA score (> 2) and *P*-values (<0.05) of the linear discriminant analysis effect size (LEfSe). Wilcoxon rank-sum test *P*-values < 0.05 are shown in orange, and those <0.01 are shown in red. The value in the square indicates the Log_10_ value of the LDA score. The results of the two tests and the mean concentration and prevalence of each metabolite are shown in Table S10. (**A**) Comparison between the periodontal disease and healthy groups; (**B**) comparison between the caries and healthy groups; (**C**) comparison between the concurrent and healthy groups. (**D, E and F**) Heat map showing metabolites with significantly higher concentrations in the healthy group when compared with each disease group. Wilcoxon rank-sum test *P*-values < 0.05 are shown in light blue, and those <0.01 are shown in blue. (**D**) Comparison between the periodontal disease and healthy groups; (**E**) comparison between the caries and healthy groups; (**F**) comparison between the concurrent and healthy groups.

We investigated changes in oral metabolites in the saliva by extracting metabolites with concentrations in the disease group that differed significantly from those in the healthy group BT, at CT, and AT. Metabolites with significantly different concentrations from those in the healthy group were selected based on the *P*-values (<0.05) of the Wilcoxon rank-sum test and LDA score (>2) and *P*-values (<0.05) of the LEfSe (19). Fig. 5A, B and C shows metabolites that are present in significantly high concentrations in the disease groups. However, Fig. 5D, E and F shows that the metabolites that were less concentrated in the disease group than in the healthy group. Some metabolites, such as threonate in the periodontal disease and concurrent groups, were consistently significantly at higher concentrations than in the healthy group from BT to AT. Some metabolites, such as histamine in the concurrent group, resolved their significant differences with the healthy group AT; however, components such as histidine (His) in the periodontal disease group showed significant differences with the healthy group AT. His was present at higher concentrations in each disease group than in the healthy group. However, other components, such as thymine, showed lower concentrations in each disease group than in the healthy group. Concentrations of UMP, uridine, thymidine, and cytidine that belong to the same pyrimidine metabolite group as thymine, were present in higher concentrations in the periodontal disease or concurrent groups than in the healthy group AT.

## DISCUSSION

In this study, the salivary microbiome and metabolome of the healthy (22 subjects), periodontal (16 subjects), caries (12 subjects), and concurrent (12 subjects) groups were compared BT , CT and AT to determine whether the oral microbiome disturbances improve after symptom remission due to dental treatment. Despite the presumed remission of symptoms from the amelioration of periodontal disease and caries-related indicators (Table 1), the salivary microbiomes of each disease group remained different from healthy subjects after dental treatment (Fig. 2A through C), with a higher relative abundance of disease-related bacteria, a lower relative abundance of nitrate-reducing bacteria such as *Neisseria* species (Fig. 3), and differences in threonate and pyrimidine metabolite concentrations observed in the periodontal disease and concurrent groups (Fig. 5). These results suggest that dysbiosis of the oral microbiome, which persists after completion of dental treatment, is associated with recurrence of oral disease after dental treatment. Here, we discuss the validity of the assumption that the samples were collected before and after dental treatment of each disease, the results of the comparison between healthy subjects and each disease group before and after dental treatment, and the differences commonly observed between healthy subjects and subjects in each disease group.

To assess microbiome disruption in patients with oral diseases, it is important to select a disease group possessing the characteristics of each disease and those of healthy subjects for comparison. The caries group had decayed teeth requiring treatment, the periodontal disease group had bleeding from PPD ≥4 mm, and the concurrent group had both. However, the healthy group had no characteristics reflecting either disease state and was not in either of the disease states for at least the last 5 years. This suggests that a suitable group of subjects was obtained for the purpose of this study. Additionally, the oral index did not worsen when compared to that at CT, even several months AT. Moreover, following the removal of the carious lesion requiring treatment, no new dental caries appeared until AT (Table 1), which indicated that disease remission occurred after dental treatment, and the oral health condition remained stable even after the transition to self-care. Therefore, it is considered that an appropriate sample was collected to evaluate remission after dental treatment.

In the periodontal disease group, we observed that the oral microbiome differs from that in healthy participants even after remission (Fig. 2A), although the relative abundance ratios of some bacterial species changed due to dental treatment (Table S7 lists the bacterial species with significant changes in relative abundance ratios due to treatment). In addition, alpha-diversity, wherein increased numerical values are associated with periodontal disease (6), remained higher than the healthy group BT and AT (Table 2). Moreover, the relative abundances of periodontal disease-related bacteria, including *P. gingivalis* and *F. alocis*, were significantly higher than those in the healthy group even after the remission of symptoms (Fig. 3A). These results suggest that the oral microbiome remains in dysbiosis with a high abundance of disease-related bacteria even after cleaning of the periodontal site. Regarding metabolome profiles, there was no significant difference in the healthy group BT (Fig. 4A). In contrast, His and threonate concentrations, which are reportedly high in patients with periodontal disease (25, 26), were significantly higher BT and AT than in the healthy group (Fig. 5A). These results suggest that, when focusing on individual components, they show differences from the healthy group that reflect the state of periodontal disease and also reflect that the condition was different from that of the healthy group even after symptom remission. In a previous study, the concentration of threonate was associated with the periodontal inflammation surface area, which quantifies the degree of gingival inflammation, and had been proposed as a candidate biomarker that reflects the severity of periodontitis (26). However, threonate concentrations in this study were high even after the remission of gingival inflammation. Therefore, it is suggested that the threonate concentration in saliva not only reflects the state of gingival inflammation improved by treatment, but also the disorder of the oral microbiome. Furthermore, for pyrimidine-related metabolites, which have been reported to be associated with periodontal disease in previous studies (9), thymine concentrations were significantly lower than in the healthy group AT (*P* < 0.01 Table S10), while uridine, thymidine, and UMP concentrations were higher at CT or AT (Fig. 5D). It has been reported that various disease-related pathogens affect host pyrimidine metabolism and alter the environment to ensure optimal pyrimidine metabolite levels to support pathogen growth (27). With regard to oral diseases, thymine is decreased and cytidine/uridine is increased in gingival fibroblasts infected with *P. gingivals* (9), and thymine is lower and uridine is higher in the gingival sulcus fluid of patients with invasive periodontitis when compared to healthy subjects (28). Therefore, the differences in pyrimidine metabolites identified in this study compared to healthy subjects may reflect that periodontal disease-associated bacteria are active in promoting an oral environment containing pyrimidine metabolites suitable for bacterial growth, suggesting that the oral microbiome undergoes dysbiosis.

In the caries group, the microbiome differed from that of healthy subjects even after decayed tooth removal and periodontal cleaning (Fig. 2B). The relative abundance of *B. dentium*, a caries-associated bacterium, was significantly higher than that in the healthy group BT, and the significant difference disappeared once at CT, but was confirmed again AT. Furthermore, the relative abundance of *S. wiggsiae*, a caries-associated bacterium, was also found to be significantly different from that in the healthy group AT (Fig. 3B). These results suggest that caries-associated bacteria still lurked in the oral cavity after dental intervention, and that the dysbiosis of the microbiome continued with a high risk of caries recurrence. On the other hand, the metabolome profile was not significantly different from the healthy group (Fig. 4B). The average number of decayed teeth was only two (Table 1), which may have made it difficult to detect differences in caries-related metabolites, such as lactate. Even in the caries group, where the percentage of BOP was lower than that in the healthy group (Table 1), the relative amounts of nine periodontal disease-associated bacteria, including the red complex bacterium *Tannerella forsythia*, were significantly higher in the caries group than in the healthy BT group (Fig. 3B), suggesting that periodontal disease bacteria may be lurking in the oral cavity even among those in the caries group with low gingival inflammation. However, AT, the proportion of periodontal indices with a PPD ≥ 4 mm, and the average PPD decreased in the caries group (Table 1), and significant differences in bacteria including *T. forsythia* from the healthy group were eliminated. This may reflect the effect of periodontal cleaning performed by a dentist prior to the treatment of dental caries.

In the concurrent group, the oral microbiome was found to be different from that of healthy individuals even after remission (Fig. 2C). Furthermore, unlike the caries and periodontal disease groups, the metabolome profile was also significantly different from the healthy group from BT to AT (Fig. 4C). In addition, alpha-diversity was higher than the healthy group BT and AT (Table 2). Moreover, the relative abundances of periodontal disease-related bacteria, including *F. alocis*, were significantly higher than those in the healthy group even after remission of symptoms and *S. wiggsiae*, a caries-associated bacterium, was also found to be significantly different from that in the healthy group AT (Fig. 3C). For metabolites, a similar trend was observed as in the periodontal group. The concentration of threonate was significantly different from that in the healthy group BT to AT in the periodontal group, and the concentration of His was also significantly different from the healthy group at CT and AT. For pyrimidine-related metabolates, we observed lower levels of thymine and higher levels of thymidine, uridine, and cytidine when compared to the healthy group AT (Fig. 5C and F). These results suggest that dysbiosis of the oral microbiome continues after caries treatment and periodontal disease remission, and that the risk of recurrence of periodontal disease and caries is high in the concomitant group. Of the three disease groups, the concurrent group had a microbiome that was more different from the healthy group than the other disease groups BT (*R^2^* value of PERMANOVA test), and only the concurrent group had a different metabolome profile when compared to the healthy group (Fig. 4C). Since there were no significant differences between the concurrent and periodontal disease groups in indices other than the number of caries (Table 1; Table S1), the differences between the concurrent and healthy groups were not due to differences in the severity of periodontal disease, but the differences in the oral microbiome and metabolome due to concurrent periodontal disease and caries. The results suggest that the degree of disruption of the microbiome is related to the characteristics of both caries and periodontal disease.

Differences in the oral microbiome from healthy subjects were not only seen in the bacterial species with high relative abundance ratios in each disease group, but also in those with low relative abundance ratios common to each disease group. The relative abundance ratio of *N. flavescens* was significantly lower in all disease groups BT when compared to that in the healthy subjects. Additionally, in *Haemophilus* and *Rothia* bacterial species, which, like *Neisseria*, are involved in intraoral nitrate reduction reactions, we found bacterial species with significantly lower abundances in the healthy group when compared to those in each disease group BT. Moreover, the relative abundance of *N. fravescens* and other nitrate-reducing bacteria AT was lower in the disease groups than in the healthy group, even after remission (Fig. 3D through F). Similar to the results of this study, the relative amounts of these nitrate-reducing bacteria have been reported to be lower in patients with dental caries and periodontal disease than in healthy subjects (29 – 31). The low relative abundance of nitrate-reducing bacteria AT is suggested to indicate continued dysbiosis of the oral microbiome. Moreover, it has been reported that the nitrate-reduction products produced by bacteria, such as *Neisseria* and *Haemophilus*, reduced the number of viable dental caries- and periodontal disease-related bacteria (32 – 34). These findings suggest that increasing the relative abundances of nitrate-reducing bacteria in the oral cavity may be important in bringing the microbiome closer to a healthy state after remission.

While establishing a feasible study, it was not without limitations. First, although the healthy group in this study had fewer filled teeth than each disease group, they had experienced dental caries and may have belonged to the disease group in the past. However, the healthy group had remained healthy for at least 5 years, and their oral microbiome showed similar characteristics to those of the healthy group shown in the previous studies, suggesting that their saliva reflected their current healthy oral condition. The difference in the microbiome between the healthy group that may have had caries in the past and the caries group immediately AT may suggest that maintaining a healthy oral condition over long term may improve dysbiosis. Notably, for some disease-related bacteria, significant differences in relative abundance with the healthy group BT were eliminated AT (Fig. 3A through C). Furthermore, the microbiome and metabolome profiles of the concurrent group resembled those of the healthy group, although there were significant differences, suggesting that even short-term maintenance of healthy condition by dental treatment is expected to show some improvement (Fig. 2F and 4F). Further follow-up of participants AT may allow us to observe the improvement in dysbiosis by keeping them free of oral disease. Second, saliva instead of dental plaque or gingival crevicular fluid was used as samples. When collecting samples from dental plaque and gingival sulcus, it is possible to determine collection sites in advance or to collect from diseased sites; however, the location of dental caries and periodontal disease varies among participants. Moreover, when dental plaque is collected from a predetermined site, it is difficult to accurately compare with healthy participants since the diseased group includes samples collected from both diseased and nondiseased sites. Furthermore, it has been reported that the bacterial flora of dental plaque differs depending on the site (35). In addition, since plaque is removed by dental treatment and appropriate self-care, we considered that a collection sample other than plaque would be appropriate to monitor; therefore, we collected mouth-rinsed water that is less burdensome for the subject and allows saliva to be collected. Although this collection method differs from the widely used saliva collection method, the microbiome data obtained with this method can be treated in the same manner as data obtained from stimulated and unstimulated saliva (16), and the metabolome studied is similar to that obtained from unstimulated saliva (17). Third, although there are concerns regarding accurate species identification in the V1-V2 region of 16S rRNA, the relative abundance ratios of individual bacterial species were compared using species-level assignment results. Since differences in the ratios of the present disease-associated species need to be assessed to evaluate whether a patient has dysbiosis, we determined that species-level comparisons were appropriate for this study. Methods are being developed that improve species-level identification by obtaining full-length 16S rRNA gene sequences, and it is expected that more accurate assessments will be made using this method. Fourth, this study is considered to be preliminary as the number of participants in each group is smaller than that in large cohort studies. When comparing the number of participants, the *P*-value of the Wilcoxon test was used as the criterion in this study, as the false discovery rate (FDR) is unlikely to detect positivity when the number of comparisons is small (FDR values described in Tables S6 and S10). Instead of using FDR, this study used LEfSe, which differs from the Wilcoxon’s test, to extract the bacterial species and metabolites detected by both methods, thereby reducing the likelihood of false positives. The results obtained in this study have many features in common with the characteristics of diseased patients and healthy participants that were described in previous reports, and the basic trends have been clarified even with the method of this study.

In conclusion, although dental treatment is effective at improving oral health conditions, it does not eliminate differences in the oral microbiome between people with oral diseases and healthy people. Dysbiosis with a high relative abundance of disease-related bacteria persists even AT. Thus, removing dental plaque in which disease-related bacteria exist by conducting regular maintenance at a dental clinic and appropriate self-care effective in removing oral biofilms (36) is important to prevent the recurrence of disease. Furthermore, reducing the risk of oral disease recurrence requires the implementation of new intervention measures to reduce the relative abundances of disease-related bacteria, which were higher than those in healthy participants, even after remission.

## MATERIALS AND METHODS

### Clinical study design

For the healthy group, we selected 22 participants among people who visited the Hiyoshi Oral Health Clinics in Sakata City, Japan, for regular maintenance and met specific criteria. The healthy group was defined as those who had no PPD ≥4 mm and no dental caries diagnosed for at least 5 years at the time of recruitment. The disease group was selected from those who visited the clinic for treatment. The caries group comprised 18 patients with dental caries requiring treatment and without bleeding from PPD ≥4 mm. The periodontal disease group comprised 24 patients with no dental caries and with bleeding from PPD ≥4 mm. The concurrent group comprised 12 patients with dental caries requiring treatment and with bleeding from PPD ≥4 mm. The exclusion criteria were as follows: (1) those with dentures (including implants), (2) smokers, (3) those who have taken antibiotics within the past 6 months, (4) those undergoing orthodontic treatment, and (5) pregnant women. This study was conducted with ethical approval from the institutional review board of the Chiyoda Paramedical Care Clinic (Chiyoda Ward, Tokyo, issue number: UMIN000031334). All participants understood the purpose of the study, and provided informed consent, and were recruited from February 2018 through January 2020, followed the treatment process and deposited samples through December 2020. Samples and data were obtained from all patients BT, but only 18 patients in the periodontal disease group, 12 in the caries group, and 12 in the concurrent group continued to visit the clinic until the end of treatment and samples were collected until AT.

Samples of the healthy group were collected upon visits to the dental clinic for regular oral maintenance. Samples of the disease groups were collected at three time points (BT, CT, and AT). Samples were collected prior to dental examinations and treatment. The CT was judged using criteria set by the dental clinic, which were as follows: PPD ≥4 mm accounted for <10%, bleeding from teeth was <10%, dental plaque index was <15%, and a good dentist’s own score calculated from home care and saliva test conditions. Ultimately, the dentist made the judgment according to the individual circumstances of the participant. Dental examinations were conducted by similarly trained dental hygienists who were employed by the same dental clinic. Periodontal examination confirmed the PPD at four locations per tooth and the presence or absence of bleeding during probing.

Treatment followed the medical treatment model (MTM), which is a comprehensive treatment, includes initial risk assessment, lifestyle instructions, such as diet and habits, and a customized maintenance program (37, 38). Treatment involved nonsurgically treating the periodontal area by scaling and root planing. In the caries group, prior to caries treatment, ultrasonic cleaning and scaling, and root planing were performed according to individual oral conditions, and the caries sites that required treatment were removed before CT. Additionally, fluoride was applied to the tooth surface at the end of each treatment in all disease groups. The remaining decayed teeth in the concurrent group at CT and AT were judged by the dentist to not require treatment and did not exhibit any problems upon follow-up observations.

The collected microbiome data and metabolite profile data of two participants was deemed incomplete in the periodontal disease group. The first sample was microbiome data acquired AT, in which one bacterial species *Delftia acidovorans* (which was present in <1% of other samples) accounted for over 50% of the relative abundance, suggesting bacterial contamination. The other sample was metabolite data at CT, and the average concentration of each metabolite in this sample was approximately 1/10th that of the samples taken from the same participant at different times (BT and AT), suggesting that the sample had been diluted before measurement. Hence, we used data from 16 participants in the periodontal disease group, excluding two participants with incomplete data.

### Sample collection

Saliva samples were collected as mouth-rinsed water (vigorously rinsed mouth with 3 mL of sterile water for 10 s). The microbiome of the mouth-rinsed water can be treated in the same way as stimulated and resting saliva microbiomes, as shown in a previous report (16). Samples were immediately stored at –80°C. Within 3 months, they were sealed in Styrofoam with dry ice and transported to the laboratory of the Lion Corporation. Upon arrival, samples were thawed at 4°C over the course of several hours, and then centrifuged (4°C, 13,000 × g for at least 5 min). After centrifugation, the pellet was used for microbiome analysis and the supernatant for metabolite analysis. It was then stored at –80°C until the next operation.

### Acquisition of microbiome data and total bacterial concentration

DNA from the sample pellet was isolated using the nexttec 1-Step DNA Isolation Kit (nexttec Biotechnologie GmbH, Leverkusen, Germany). Polymerase chain reaction (PCR) was performed by adjusting the amount of DNA added to <20 µg based on the DNA concentration measured by Qubit 4 Fluorometer (Thermo Fisher Scientific, MA, USA). PCR was performed using universal primers (27Fmod and 338R) for sequencing of the 16S rRNA gene V1-V2 region, as previously described (16). Ex Taq polymerase (Takara Bio, Shiga, Japan) was used for amplification, after which the PCR products were purified, pooled based on concentrations quantified with the Quant-iT PicoGreen dsDNA Assay Kit (Thermo Fisher Scientific, MA, USA), and curated into a library. The DNA library was sequenced using MiSeq Reagent Kit V3 (300  ×  2 cycles) and a MiSeq sequencer (Illumina, San Diego, CA, USA). Reads with an average quality value (QV)  <25 and those lacking the primer sequences at both ends were filtered off. Possible chimeric sequences that had alignment lengths of  <90% coverage with authentic reference 16S rRNA gene sequences in the database were also removed. After trimming both primer sequences from the filter-passed reads, 10,000 reads per sample were randomly selected and grouped into OTUs using the UCLUST algorithm (version 5.2.32) with a 97% identity threshold. All recruited participant data, including participants for whom AT data were not collected, were used to group the OTUs. Taxonomic assignments for each OTU were made by similarity searching against a HOMD 16S rRNA RefSeq database (version 15.22) using the GLSEARCH program (version 36.3.8 g). For assignment at the species levels, sequence similarity thresholds of 97% were applied.

Quantitative PCR was performed to determine the total bacterial concentration in each sample. We performed quantitative PCR using the extracted genomic DNA as the sample DNA template with each primer and probe. The primer and probe sequences (5′ to 3′) were as follows: forward primer, TCCTACGGGAGGCAGCAGT; reverse primer, GGACTACCAGGGTATCTAATCCTGTT; probe, FAM-CGTATTACCGCGGCTGCTGGCAC-TAMRA (39). The reaction mixture was analyzed using the CFX Connect system (Bio-Rad Laboratories Inc., Hercules, CA, USA). We calculated the total bacterial concentration (estimated CFU/mL) referring to the experimental method (40). The concentration of total bacteria from the samples was calculated using standard curves, which were generated by plotting the threshold cycle (CT) values against the bacterial concentration (CFU/mL). Standard curves were used with serial dilutions of known amounts of *P. gingivalis* ATCC 33277 DNA.

### Acquisition of metabolome data

The supernatant, after initial centrifugation, was transferred to a 5 kDa cutoff filter (Human Metabolome Technologies, Tsuruoka, Japan) to remove proteins >5 kDa. Prior to capillary electrophoresis–time-of-flight mass spectrometry (CE–TOFMS) analysis, the filtrate was centrifuged at 40°C for approximately 3 h using a Refrigerated CentriVap Concentrator (LABCONCO, Kansas City, USA) and dried. To the dry solids, 60 µL of Milli-Q water-containing reference compounds (200 µM each of methionine sulfone, D-camphor-10-sulfonic acid, 3-aminopyrrolidine, and trimesic acid) was added. CE-TOFMS-based metabolome profiling was performed using an Agilent 7100 Capillary Electrophoresis system (Agilent Technologies, Waldbronn, Germany), Agilent 6224 TOF LC/MS system (Agilent Technologies, Santa Clara, CA), Agilent 1200 series isocratic HPLC pump, G1603A Agilent CE-MS adapter kit, and G1607A Agilent CE-electrospray ionization-MS sprayer kit, as previously described (17). Data processing was performed using the metabolome analysis software MasterHands (41, 42). Quality control (QC) samples were mixed with supernatant samples from multiple participants in each group and assessed simultaneously for each set of measurements. One hundred and fifty-five metabolites were detected in QC samples for each measurement. Based on the concentration values in the QC samples, those of the 155 components in each sample were adjusted for batch-to-batch errors.

### Data analysis

All statistical test results were obtained using the R software version 4.04 (43) and *P*-values for each statistical test are shown in Tables S2 to S11. Wilcoxon test was performed using exactRankTests package version 0.8–34. PERMANOVA test, and Shannon diversity index calculations were performed using the vegan package version 2.5–7. Steel–Dwass test was performed using NSM3 package version 1.16. In addition to the Wilcoxon test, a LEfSe (19) was used to extract bacterial species and metabolites with significantly different abundance ratios and concentrations from the healthy subjects. From the estimated effect size of significant features, only those features with scaled LDA analysis scores above the threshold score of 2.0 and *P*-values <0.05 were labeled as differentially abundant. To compare the microbiomes, we calculated the weighted UniFrac distance, which is used to compare biological communities accounting for phylogenetic distances between observed organisms (44), by OTUs and their representative sequences created with 97% homology using 10,000 reads per sample. To compare the metabolite profiles, we calculated Bray–Curtis dissimilarity, which is used to quantify the compositional dissimilarity between two different samples (45), using the concentrations of 155 metabolites.

## Data Availability

The microbiome analysis data (16S rRNA gene sequences) have been deposited in the DNA Data Bank of Japan (accession number DRA015381). Subject data collected in this study, such as oral examination and metabolome data sets generated during and/or analyzed during the current study are available from the corresponding author on reasonable request. A STORMS (Strengthening the Organizing and Reporting of Microbiome Studies) (46) checklist is available at Zenodo DOI 10.5281/zenodo.8166497.

## SUPPLEMENTAL MATERIAL

Supplemental tables msystems.00683-23-s0001.xlsx Tables S1 to S11.

ASM does not own the copyrights to Supplemental Material that may be linked to, or accessed through, an article. The authors have granted ASM a non-exclusive, world-wide license to publish the Supplemental Material files. Please contact the corresponding author directly for reuse.
